# Supplementary material for: Modeling glioblastoma heterogeneity as a dynamic network of cell states
Source: Mol Syst Biol. 2021 Sep 16;17(9):e10105. doi: 10.15252/msb.202010105 (PMC8444284; doi:10.15252/msb.202010105)
Supplement: Supplementary file 6 — Source Data for Figure 5 [file MSB-17-e10105-s004.zip › Figure5A_sourcedata/GSEA_3071/nosigdb_stateVI.GseaPreranked.1622795169056/DOROSZKO_U3054MG_CLUSTER_0.html]

Details for gene set DOROSZKO\_U3054MG\_CLUSTER\_0[GSEA]

|  || Dataset | state63071 |
| Phenotype | NoPhenotypeAvailable |
| Upregulated in class | na\_neg |
| GeneSet | DOROSZKO\_U3054MG\_CLUSTER\_0 |
| Enrichment Score (ES) | -0.6192266 |
| Normalized Enrichment Score (NES) | -2.6359704 |
| Nominal p-value | 0.0 |
| FDR q-value | 0.0 |
| FWER p-Value | 0.0 |
Table: GSEA Results Summary

  

Fig 1: Enrichment plot: DOROSZKO\_U3054MG\_CLUSTER\_0      
 Profile of the Running ES Score & Positions of GeneSet Members on the Rank Ordered List

  

| PROBE | GENE SYMBOL | GENE\_TITLE | RANK IN GENE LIST | RANK METRIC SCORE | RUNNING ES | CORE ENRICHMENT || 1 | ALCAM |  |  | 7 | 1.185 | 0.0279 | No |
| 2 | ETS2 |  |  | 52 | 0.864 | 0.0374 | No |
| 3 | MGLL |  |  | 190 | 0.609 | 0.0146 | No |
| 4 | SFRP1 |  |  | 426 | 0.479 | -0.0388 | No |
| 5 | L1CAM |  |  | 971 | 0.350 | -0.1814 | No |
| 6 | ARHGEF16 |  |  | 1021 | 0.341 | -0.1864 | No |
| 7 | KHDRBS3 |  |  | 1313 | 0.302 | -0.2598 | No |
| 8 | BMPER |  |  | 1776 | 0.253 | -0.3820 | No |
| 9 | MAPT |  |  | 1922 | -0.267 | -0.4156 | No |
| 10 | TMEM132B |  |  | 2168 | -0.308 | -0.4761 | No |
| 11 | UCHL1 |  |  | 2232 | -0.320 | -0.4856 | No |
| 12 | SEMA6D |  |  | 2377 | -0.349 | -0.5169 | No |
| 13 | COL15A1 |  |  | 2379 | -0.349 | -0.5083 | No |
| 14 | SAP30 |  |  | 2413 | -0.356 | -0.5086 | No |
| 15 | TRIM2 |  |  | 2481 | -0.374 | -0.5178 | No |
| 16 | GLTP |  |  | 2653 | -0.412 | -0.5550 | No |
| 17 | ADAM23 |  |  | 2682 | -0.420 | -0.5522 | No |
| 18 | CHRDL1 |  |  | 2735 | -0.434 | -0.5558 | No |
| 19 | AP002884.1 |  |  | 2937 | -0.509 | -0.5989 | No |
| 20 | PDLIM3 |  |  | 3011 | -0.546 | -0.6055 | Yes |
| 21 | SLC44A1 |  |  | 3013 | -0.546 | -0.5920 | Yes |
| 22 | FABP5 |  |  | 3079 | -0.577 | -0.5956 | Yes |
| 23 | TNFAIP6 |  |  | 3085 | -0.578 | -0.5824 | Yes |
| 24 | SHC3 |  |  | 3093 | -0.581 | -0.5697 | Yes |
| 25 | ERBB3 |  |  | 3116 | -0.596 | -0.5608 | Yes |
| 26 | OLIG1 |  |  | 3126 | -0.601 | -0.5482 | Yes |
| 27 | MCAM |  |  | 3208 | -0.676 | -0.5537 | Yes |
| 28 | SOX2 |  |  | 3314 | -0.778 | -0.5634 | Yes |
| 29 | PMEPA1 |  |  | 3338 | -0.804 | -0.5495 | Yes |
| 30 | PRUNE2 |  |  | 3348 | -0.810 | -0.5316 | Yes |
| 31 | SLAIN1 |  |  | 3385 | -0.868 | -0.5198 | Yes |
| 32 | BCHE |  |  | 3405 | -0.909 | -0.5022 | Yes |
| 33 | CD9 |  |  | 3445 | -0.995 | -0.4880 | Yes |
| 34 | PLCB1 |  |  | 3468 | -1.045 | -0.4678 | Yes |
| 35 | TTYH1 |  |  | 3481 | -1.067 | -0.4443 | Yes |
| 36 | NCAM1 |  |  | 3514 | -1.168 | -0.4238 | Yes |
| 37 | SOX6 |  |  | 3517 | -1.174 | -0.3948 | Yes |
| 38 | PCSK1N |  |  | 3522 | -1.197 | -0.3658 | Yes |
| 39 | TUBB2B |  |  | 3532 | -1.228 | -0.3374 | Yes |
| 40 | CDH2 |  |  | 3543 | -1.297 | -0.3075 | Yes |
| 41 | CKB |  |  | 3579 | -1.575 | -0.2776 | Yes |
| 42 | CADM1 |  |  | 3581 | -1.591 | -0.2378 | Yes |
| 43 | FAM181B |  |  | 3582 | -1.594 | -0.1977 | Yes |
| 44 | SORL1 |  |  | 3609 | -1.895 | -0.1572 | Yes |
| 45 | COL9A3 |  |  | 3618 | -2.307 | -0.1014 | Yes |
| 46 | PTPRZ1 |  |  | 3637 | -4.237 | 0.0003 | Yes |
Table: GSEA details [plain text format]

  

Fig 2: DOROSZKO\_U3054MG\_CLUSTER\_0: Random ES distribution      
 Gene set null distribution of ES for **DOROSZKO\_U3054MG\_CLUSTER\_0**
